# The Role of the Microbiome in the Metabolic Health of People with Schizophrenia and Related Psychoses: Cross-Sectional and Pre-Post Lifestyle Intervention Analyses

**DOI:** 10.3390/pathogens11111279

**Published:** 2022-11-01

**Authors:** Maryanne O’Donnell, Scott B. Teasdale, Xin-Yi Chua, Jamie Hardman, Nan Wu, Jackie Curtis, Katherine Samaras, Patrick Bolton, Margaret J. Morris, Cyndi Shannon Weickert, Tertia Purves-Tyson, Fatima El-Assaad, Xiao-Tao Jiang, Georgina L. Hold, Emad El-Omar

**Affiliations:** 1 Discipline of Psychiatry and Mental Health, School of Medicine and Health, University of New South Wales, Kensington 2033, Australia; 2 Eastern Suburbs Mental Health Service, South Eastern Sydney Local Health District, Randwick 2031, Australia; 3 Mindgardens Neuroscience Network, Sydney 2033, Australia; 4 Microbiome Research Centre, St George and Sutherland Clinical Campuses, University of New South Wales, Kogarah 2217, Australia; 5 Department of Gastroenterology, The Sutherland Hospital, Caringbah 2229, Australia; 6 Department of Endocrinology, St Vincent’s Hospital Sydney, Victoria St, Darlinghurst 2010, Australia; 7 Clinical Obesity, Nutrition and Adipose Biology Lab, 384 Garvan Institute of Medical Research, Victoria St, Darlinghurst 2010, Australia; 8 School of Clinical Medicine, St Vincent’s Healthcare Clinical Campus, University of New South Wales, Darlinghurst 2010, Australia; 9 School of Public Health, University of New South Wales, Kensington 2033, Australia; 10 School of Medical Sciences, University of New South Wales, Kensington 2033, Australia; 11 Schizophrenia Research Laboratory, Neuroscience Research Australia, Sydney 2033, Australia; 12 Department of Neuroscience and Physiology, Upstate Medical University, Syracuse, NY 13210, USA

**Keywords:** microbiota, antipsychotic agents, schizophrenia, lifestyle, metabolic diseases

## Abstract

The microbiome has been implicated in the development of metabolic conditions which occur at high rates in people with schizophrenia and related psychoses. This exploratory proof-of-concept study aimed to: (i) characterize the gut microbiota in antipsychotic naïve or quasi-naïve people with first-episode psychosis, and people with established schizophrenia receiving clozapine therapy; (ii) test for microbiome changes following a lifestyle intervention which included diet and exercise education and physical activity. Participants were recruited from the Eastern Suburbs Mental Health Service, Sydney, Australia. Anthropometric, lifestyle and gut microbiota data were collected at baseline and following a 12-week lifestyle intervention. Stool samples underwent 16S rRNA sequencing to analyse microbiota diversity and composition. Seventeen people with established schizophrenia and five people with first-episode psychosis were recruited and matched with 22 age-sex, BMI and ethnicity matched controls from a concurrent study for baseline comparisons. There was no difference in α-diversity between groups at baseline, but microbial composition differed by 21 taxa between the established schizophrenia group and controls. In people with established illness pre-post comparison of α-diversity showed significant increases after the 12-week lifestyle intervention. This pilot study adds to the current literature that detail compositional differences in the gut microbiota of people with schizophrenia compared to those without mental illness and suggests that lifestyle interventions may increase gut microbial diversity in patients with established illness. These results show that microbiome studies are feasible in patients with established schizophrenia and larger studies are warranted to validate microbial signatures and understand the relevance of lifestyle change in the development of metabolic conditions in this population.

## 1. Introduction

Schizophrenia and related psychoses are serious psychiatric disorders with uncertain etiology that disrupt multiple life domains and may persist causing significant disease burden [1]. People living with schizophrenia and related psychotic disorders face a 15-year shortfall in life-expectancy compared to people in the general population [2]. Concomitant diseases caused by cardiometabolic derangement are the biggest contributor to premature death, with an 85% higher risk of death from cardiovascular disease compared to the general population [3]. The reasons for such a disparity in physical health conditions is not fully understood but appear to be multifaceted and are frequently linked to side effects of antipsychotic medication which drive increased appetite, rapid weight gain and metabolic derangement [4], poor diet quality [5], and low physical activity [6].

Gut microbiota dysbiosis is implicated in the development of conditions such as obesity and diabetes in the general population [7,8]. Studies of people with schizophrenia and the microbiota yield inconsistent findings, although there is support for compositional differences in taxa between healthy controls and patients [9,10,11,12]. Whether these changes are primary to the illness itself, or secondary to treatment effects and associated metabolic changes, requires investigation. Antipsychotic medications, the primary pharmacological treatment for psychosis, have a profound impact on the microbiota [13], though how that relates to the development of metabolic complications is unclear. Obesity induces a low-grade inflammatory state with elevated pro-inflammatory cytokine levels [14], and immune activation is implicated in the development of schizophrenia [15,16]. The relationship between the microbiota, inflammation, and schizophrenia and how these can be modulated by antipsychotics and/or exercise and diet changes is not established [17].

Addressing metabolic derangements and reducing disease burden through diet modification, exercise, and pharmacotherapy is well understood in populations without mental illness [18,19]. Application of these interventions in people experiencing schizophrenia and related mental illness remains challenging [20]. For example, barriers to accessing lifestyle interventions as well as illness factors such as reduced motivation, are common for people with established schizophrenia [21], and thus reduces ability to participate in such behavioral change interventions.

Evaluation of the physical health of people engaged in an early psychosis program, a clozapine clinic and a long-acting injectable antipsychotic treatment clinic, within a public mental health service, in Sydney, Australia, found high rates of metabolic syndrome, physical comorbidities, and detrimental lifestyle [22,23,24]. Curtis et al. (2016) demonstrated the capacity to mitigate the impact of antipsychotic medications on weight change in people with first-episode psychosis early in the course of treatment with an intensive lifestyle and life-skills program—the Keeping the Body in Mind program (KBIM). This innovative intervention, utilizing exercise physiologists and dieticians working closely with mental health clinicians, focuses on psychoeducation, diet, exercise, and close monitoring of metabolic markers [25]. Participants of the 12-week KBIM intervention gained significantly less weight (1.8 kg, 95% CI, −0.4 to 2.8 vs. 7.8 kg, 4.8 to 10.7, *p* < 0.001), with similar effects for waist circumference and indicators of cardiometabolic risk. After 12-weeks, participants could continue accessing elements of the KBIM program, however, intensity of follow-up by KBIM clinicians was often reduced and dependent on cardio-metabolic risk and level of desire of the participant. Two-year follow-up of KBIM participants found the intervention prevented the significant weight gain usually observed on antipsychotic medication [26]. Subsequently the KBIM program was scaled up and delivered to other high-risk population groups within the public mental health service such as those receiving clozapine therapy, an antipsychotic medication used for treatment resistant schizophrenia. This provided a unique opportunity to investigate whether dysbiosis in the gut microbiota may underpin metabolic dysfunction in patients treated with antipsychotics and evaluate the role of the KBIM lifestyle intervention on gut microbiota measures as a potential mediating factor in improving metabolic health.

This exploratory proof of concept study aimed to test the feasibility of gut microbiota investigation in people with schizophrenia and related psychoses and explore whether there is a potentially modifiable relationship between the use of antipsychotic medication, gut microbiota and the metabolic syndrome.

The main objectives were:

(i) To conduct a pilot study to characterize the gut microbiota in two patient groups, those with first-episode psychosis and those with established schizophrenia, compared with matched controls.
(ii) To examine gut microbiota diversity and composition before and after treatment with lifestyle intervention in enhanced clinical settings.

## 2. Methods

### 2.1. Study Design and Setting

This mixed design preliminary study included cross-sectional and prospective analyses. The study was carried out through inpatient and community services of the Eastern Suburbs Mental Health Service, South Eastern Sydney Local Health District (SESLHD). Ethics approval was obtained from the SESLHD Human Research Ethics Committee (#HREC 18 202; 2019/ETH11443). Reporting followed the Strengthening the Reporting of Observational Studies in Epidemiology (STROBE) statement [27].

### 2.2. Participants

Three groups of participants were recruited:

#### 2.2.1. Group 1. First Episode Psychosis

Patients diagnosed with a first-episode psychosis, by a clinical psychiatrist, aged between 18–25 years, who were antipsychotic naïve or within the first 4 weeks of treatment with antipsychotic medication, and attending the community based early psychosis program, or inpatient mental health facilities, and who were offered participation in the KBIM program.

#### 2.2.2. Group 2. Established illness (Patients on Clozapine)

Patients between 18–65 years, with an established clinical diagnosis of schizophrenia/schizoaffective disorder (collectively called schizophrenia) who had been on clozapine medication for at least 1 year, and were participating in the KBIM Program, were recruited from the clozapine clinic, where the KBIM Program is established as an integral component of usual care.

Mental health exclusion criteria in both groups included patients who had marked communication difficulties because of language, mental illness or cognitive impairment, or meeting DSM-5 criteria for severe substance abuse disorder, and alcohol abuse disorder. Patients receiving more than one antipsychotic medication were not excluded from the sample.

#### 2.2.3. Group 3. Matched Controls (MCs)

Controls were people without a mental illness that were individually matched on age, sex, ethnicity, and BMI with each of the subjects in group 1 and 2, hereafter referred to as matched controls. Matched controls were excluded if they reported any history of physical illness (metabolic syndrome, Type 2 diabetes, hypertension, dyslipidemia, autoimmune disorders or current infection). Treatment with medication, including antipsychotics, oral hypoglycemics, insulin, antihypertensives, statins, other psychoactive medications, and antibiotics, pre and probiotic therapies and omega 3 fatty acids during the last 3 months, were also exclusion criteria.

Matched controls were drawn from two sources: (i) the Neuroscience Research Australia volunteers’ database and (ii) the Australian IBD Microbiome study (AIM). Matched control samples were collected during the same time interval and from within the same geographic region to minimize experimental disparity.

### 2.3. Sample Size

This pilot study aimed to recruit 20 participants for each of the three groups: (i) first-episode psychosis, (ii) established illness, and (iii) matched controls, based on the available funding and the capacity of the study team to conduct a proof of concept, feasibility pilot study.

Unfortunately, recruitment for the study was interrupted due to the imposition of COVID-19 restrictions 6 months into data collection, with cessation of all non-essential contacts with the health service. This impacted data collection from each of the groups in different ways. The most affected was the first-episode psychosis group who were slow to recruit prospectively due to the acuity of their mental states affecting their capacity to participate in the study and provide informed consent. Consequently only 5 subjects were recruited.

The second group of participants, people with established illness, were recruited from the clozapine clinic, a well-established clinic with over 160 people with enduring schizophrenia, attending on a 3 monthly basis. Participants in this group were invited to participate by their treating clinicians as they presented for their 3 monthly reviews. With COVID-19 restrictions imposed, the clinic was transformed into a tele-clinic, severely impacting on attendance at the clinic with recruitment yielding only 17 participants.

### 2.4. Procedure

All patients were referred to the study by their treating clinicians who continued to manage their care with respect to the prescription of psychotropic and cardio-metabolic medications as well as psychosocial support. Participants completed written informed consent prior to commencing the study. Patients with active or residual psychotic symptoms underwent capacity assessment. Clinical and demographic details were obtained by the research officer from medical records and included: age, sex, ethnicity, psychotropic medication prescription, length of exposure to clozapine, and metformin prescription. Routine metabolic and research bloods were collected from participants within 72 h of stool collection.

Participants in both study groups provided blood and stool samples at baseline on entry to the study, then continued with medical ‘treatment as usual’ and participated in the KBIM program over 12 weeks, after which second samples of blood and stool were collected. Stool samples of the control group were only available for baseline comparisons.

The KBIM program included individualized and group components delivered by a specialist team embedded within the community mental health service, and comprised a clinical nurse consultant, dietitian, exercise physiologist and peer worker (person with lived experience of mental illness). Intervention elements included individualized consultations/health coaching offered weekly, onsite gym with specialist support open five days per week, and cooking and sports groups were offered weekly. Information concerning diet, exercise and metabolic parameters were obtained at baseline and post 12-week intervention from their medical records. The extent of participation in the program was measured by the number of weekly attendances participants had with the KBIM team. KBIM clinicians entered anthropometric and metabolic data into the medical file in line with treatment as usual. The study research officer collected the data from the medical record.

Matched controls for each of the participants in the patient groups were then identified. First, the Neuroscience Research Australia database which offered options for participation to registrants based on age, sex and non-psychiatric illness, was searched. The research officer could then screen them for metabolic and autoimmune illness prior to them attending for medical review and stool and blood collection. This process yielded only 5 subjects prior to imposition of COVID restrictions. Second, the Microbiome Research Centre (MRC) offered an alternative source of matched controls from a concurrent large study, the Australian IBD Microbiome (AIM) study [28], which had a control database of over 200 subjects with access to stored stool samples, in association with demographic, metabolic, dietary and ethnicity data. Ethics approval was obtained to approach participants of the AIM study and seek consent for their data to be utilized as match controls in the current study (2019/ETH11443).

The procedure for data collection was consistent for patient and matched control groups, however, the person taking the measures, e.g., for anthropometric measures, differed between patient groups and the matched control group.

### 2.5. Blood Collection

Blood (~5 mL per tube) were collected by the Prince of Wales Hospital Pathology Service into enthylenediaminetetraacetic acid (EDTA; for plasma), serum gel (for serum) and acid citric dextrose (ACD, for RNA) tubes (Interpath, Somerton, Australia) and processed within 30 min at NeuRA using standard protocols. Plasma, serum and RNA are stored at −80 °C.

### 2.6. Stool Collection and Sequencing

A short video was shown to participants demonstrating how to collect samples. Stool samples were collected by participants using a ColOff^®^ Specimen Collection Facilitator Device (ColOff^®^ Industrial, Brazil), then scoop collected stool into PSP^®^ Stool Collection Tube containing DNA Stabilizer (STRATEC Molecular; Thermofisher, Invitek, Germany). All samples were stored at −80 °C within 72 h of collection.

Total DNA was extracted from stool samples using the PSP^®^ Spin Stool DNA Plus Kit (STRATEC Molecular). The V3-V4 hypervariable region of the 16S rRNA gene was targeted for amplicon sequencing using the 341f-805r primer pair [29]. Samples were sequenced on Illumina MiSeq, generating paired end 300bp reads. Sequencing data were processed following the QIIME2 (2020.8 release via conda) pipeline [30], where data were denoised, dereplicated and filtered for chimeric reads using DADA2 [31], then to generate amplicon sequence variants (ASVs). Each ASV was assigned to a taxon using a naïve bayes classifier trained on the V3–V4 hypervariable region of reference sequences from the Greengenes database (release 13_5) [32]. Data were normalized by rarefaction to a sample depth of 30,000 reads per sample.

### 2.7. Explanatory Outcomes and Clinical/Demographic Details

Metabolic parameters were measured by KBIM program clinicians and included body weight (kg), BMI (kg/m^2^), and waist circumference (cm). Diet quality was measured through dietary assessments conducted by a KBIM dietitian (diet history or 3-day prospective food record) and analyzed through FoodWorks 9 (Xyris Pty Ltd., Brisbane, Australia) and the Dietary Guidelines Index (DGI-2013) score was calculated [33]. Higher diet quality scores equate to greater adherence to dietary guidelines and reduced risk of cardiometabolic complications. VO2-submax was measured via the Astrand 6-min cycle ergometer test [34], facilitated by a KBIM exercise physiologist. VO2-submax is a measure of cardiovascular fitness an independent risk factor for cardiometabolic complications.

Controls were screened for physical illness and treatment with medication by the research officer attached to the AIM Study and by the research officer associated with the Neuroscience Research Australia database. BMI was measured and data relating to ethnicity and diet were collected. Data was only collected on matched controls at baseline and only compared to baseline samples for both groups of subjects.

### 2.8. Data Analysis

Between group statistical comparisons (total with psychosis and controls) were performed on baseline and pre-post measures using Chi-Squared for categorical variables (sex, ethnicity, and BMI classification), paired sample t-tests for normally distributed continuous variables (weight, BMI, and diet quality) and Wilcoxon Signed Rank Test for non-normally distributed continuous variables (age and VO2 submax). α-diversity analyses were assessed using different α-diversity metrics returned by otuSummary (v0.1.1) R package (incl. Chao1, Chao2, Evenness, Gini, Invsimpson, ObservedFeatures, Shannon, and Simpson). We tested for group differences using paired samples Wilcoxon Signed Rank Test comparing control-vs-psychosis, control-vs-established, and control-vs-first-episodes. We additionally compared if there were any difference across the three groups using Kruskal-Wallis test. Correlation analysis between α-diversity and explanatory outcomes was performed using repeated-measures correlation implemented in the rmcorr (v0.5.0) R package [35]. Tests for compositional differences as summarised by β-diversity metrics were performed using the Permutational Multivariate Analysis of Variance (PERMANOVA) method [36], implemented in the adonis2 function in vegan (v2.5.7) [37]. Detection of differentially abundant taxa was carried out using Linear discriminant analysis Effect Size (LEfSe, v1.1.2 via conda) [38]. Plots were generated using ggplot2 in R [39].

## 3. Results

### 3.1. Baseline

There were 44 participants recruited, aged 18–65 years: five with a first-episode psychosis, 17 with established illness on long term clozapine medication, and 22 matched controls. Mean clozapine exposure in the established illness group was 10.8 ± 7.5 years. There were no statistically significant differences in age, sex, or BMI between those with psychotic illness and the matched controls at baseline (Table 1).

**Figure 1:**
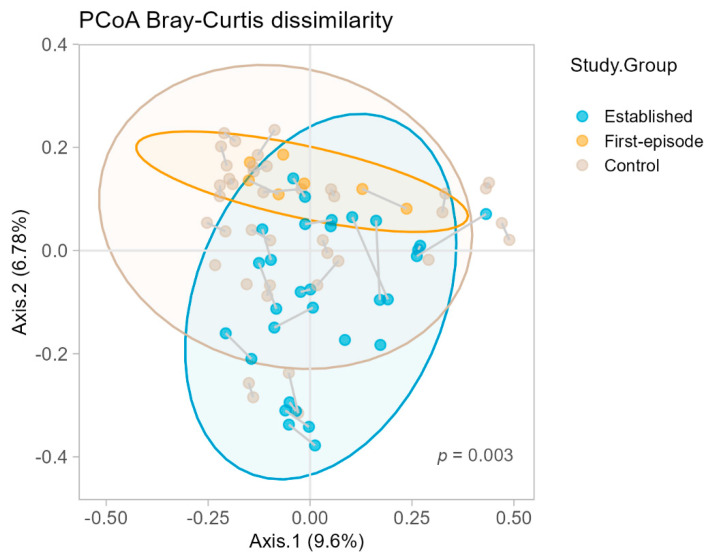
Principal coordinate analysis plot showing the Bray-Curtis dissimilarity between the three study groups, matched controls, established illness and first-episode psychosis. The permutational multivariate analysis of variation (PERMANOVA) test returned *p* = 0.003. Further pairwise comparisons showed differences between controls and established illness (*p* = 0.003), established illness and first-episode psychosis (*p* = 0.006) but no difference between matched controls and first-episode psychosis (*p* = 0.204).

**Figure 2:**
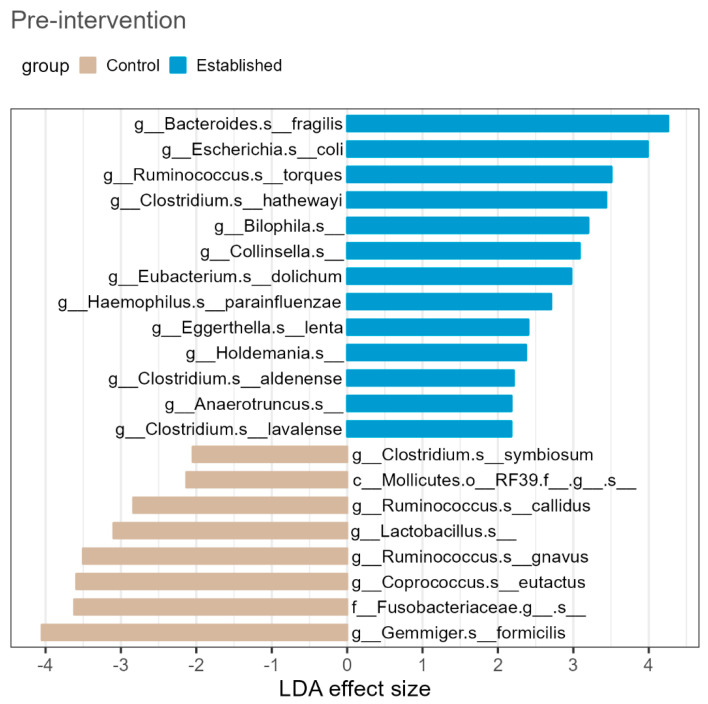
There were 21 taxa identified as differentially abundant between matched controls and established illness, using LEfSe analysis, pre lifestyle intervention. Blue bars on the right-hand of the zero-line indicate taxa that are higher in relative abundance in the established illness group compared to matched controls, while grey bars on the left-side of the zero-line indicate taxa that are lower in relative abundance in established illness group compared to matched controls.

In cross-sectional comparisons, there were no statistically significant differences detected in overall stool microbial α-diversity across any of our group comparisons (Supplementary Figure S1). However, there were significant compositional differences (β-diversity) between the three groups; measured by PERMANOVA using Bray-Curtis dissimilarity (Figure 1). Pairwise PERMANOVA comparisons showed differences for matched controls versus established illness (*F* [1] = 2.73, *p* = 0.003), and for established illness versus first-episode psychosis (*F* [1] = 2.23, *p* = 0.006). There was no significant difference between matched controls and first-episode psychosis (*F* [1] = 1.4, *p* = 0.204). LEfSe analysis showed that 21 taxa were differentially abundant between matched controls and those with established illness (Figure 2).

### 3.2. 12-Week Follow-Up

Post lifestyle intervention (12-weeks), stool samples were collected on all 17 people within the established illness group, however, after rarefaction normalization, 14 remained. Of these 14 people, additional pre and post-intervention measures were available for the following: weight (n = 12), BMI (n = 12), diet quality (n = 12) and VO2 submax (n = 8). Post-intervention stool samples were collected on two people with first-episode psychosis. No further analyses were conducted on the first-episode group due to insufficient data.

The mean number of lifestyle intervention sessions attended in the established illness group was 14.0 ± 8.1 sessions. There were no statistically significant differences in weight (mean difference [*MD*] = 0.3 kg ± 2.2, *t* (12) = 0.42, *p* = 0.68), BMI (*MD* = 0.1 kg/m^2^ ± 0.7, *t* (12) = 0.28, *p* = 0.78), diet quality (*MD* = 8.3 ± 26.3, *t* (11) = 1.10, *p* = 0.30), or VO2 submax (*MD* = 2.0 ± 7.5, *Z* = 11, *p* = 0.67) from baseline to follow-up.

**Figure 3:**
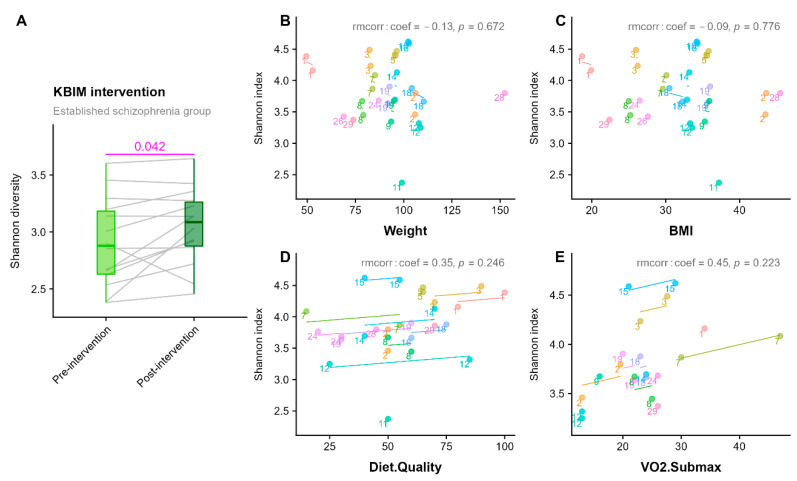
(**A**) Significant increase (*p* = 0.042) in gut microbiota, α-diversity measured by Shannon index, from pre-to-post lifestyle intervention for the established illness group. Grey lines join samples from the same patient. (**B**–**E**) Repeated-measures correlations (measured using rmcorr R package) between the α-diversity (Shannon index) and clinical measures. No correlation is statistically significant, however, the directions of the associations were of interest.

There was an increase in α-diversity measured by Shannon index following the lifestyle intervention (n = 14, *MD* = 0.14 ± 0.24, *Z* = 20, *p* = 0.042) (Figure 3A and Supplementary Figure S2). Visual inspection of correlation analyses suggests a potential positive relationship between diet quality and α-diversity; however, this was not statistically significant (Figure 3D). There was no statistically significant difference in β-diversity or species abundance pre-post lifestyle intervention in the established illness group.

## 4. Discussion

This pilot study demonstrates that: (i) microbial composition differs by 21 taxa in people with schizophrenia receiving clozapine therapy compared to controls without a mental illness, matched for age, sex, ethnicity and BMI; and (ii) microbiota diversity of people with schizophrenia on clozapine therapy can be increased through a 12-week lifestyle intervention.

A difference in the microbial composition but not in α-diversity in patients with schizophrenia compared to controls, is consistent with the broader literature. A 2022 systematic review of observational studies explored differences in α- and β-diversity between people with schizophrenia and controls without mental illness [9]. Fifty-one of the 63 analyses (81%) comparing α-diversity found no difference between groups, while 15 of 19 analyses (79%) found a significant difference in β-diversity, although the taxa reported to differ between groups in the systematic review were not consistent across all studies or with the taxa in this study [9,10,11,12]. Zhu et al. (2020) in a large comprehensive metagenome-wide association study (MWAS) of medication free patients, identified 11 different taxa, including *Akkermansia*, *Bifidobacterium* and *Streptococcus vestibularis*, to be significantly enhanced in patients with schizophrenia compared to healthy controls [40]. At the genus level they noted overlap in 6 genera with Zheng et al. (2019) [41] and to a lesser extent with Shen et al. (2018) [42]. We cannot report any overlap with the bacteria identified by these research groups, however, our population of patients were at different stages of their illness, on long-term clozapine therapy, had much higher BMIs than the patients in these studies and were from a different geographic region and of mixed ethnicity.

While comparison of taxa reported in other studies may benefit from consistency in reporting at both phyla and genus level there has been some consensus that of the 4 major phyla represented in the microbiome (Firmicutes, Bacteroidetes, Actinobacteria and Proteobacteria), a healthier microbiome is characterized by more Firmicutes and that a high fat diet is more likely to be overrepresented by Proteobacteria [43,44]. In our study, patients with established illness demonstrated an increase in abundance of the Proteobacteria; *Bilophila*, *Collinsella* and *Haemaophilius* and species belonging to the Firmicutes phyla, which is known to have a beneficial effect on gut maintenance and homoeostasis were underrepresented, ie *Lactobacillus*, *Fusobacteria*, *Ruminococcos* and *Copracoccus*. Of note is the abundance of *Bilophila* in established illness compared to the matched controls. This hydrogen sulfide producing Proteobacteria has been identified in diets high in animal fats and excessive dairy to increase the secretion of bile acids which feed *Bilophila* and to decrease the production of short chain fatty acids (SCFAs) including butyrate in mice studies [45]. *Bilophila* has also been implicated in systemic inflammatory disease including Irritable Bowel Disease (IBD) in mice and humans [46,47]. McGuiness et al. (2022) suggest we need greater understanding of the clinical or functional meaning of these differences, as it remains unclear whether they reflect disruptions in physiological processes [9].

An increase in α-diversity, post the KBIM intervention is consistent with expectations that lifestyle changes would increase the abundance and diversity of the microbiome. A likely mechanism is that an increase in diet quality reflects an increase in the intake of nutrient rich foods high in fiber, such as wholegrains, fruit and vegetables. However, these findings need to be interpreted with caution, given there were no significant differences in α-diversity between people with established illness and their matched controls at baseline. In this regard the effect of obesity itself on the microbiome may be relevant [44]. An advantage of our study, despite the small sample size, was the capacity to match our participants on BMI and ethnicity. This included controls whose BMIs were in the obese range, perhaps negating any α-diversity effects that may have been attributed to obesity itself.

In the pre-posttest paradigm, the change noted was relative to the subjects themselves. Laitinen and Mokkala’s (2019) findings showing a significant relationship between dietary quality and α-diversity, measured by the Shannon index [48], may indicate that although our findings, relating to diet quality did not reach significance, the positive direction of the association suggests a study with a larger sample size might clarify the contribution of diet quality to α-diversity enrichment.

Dietary and other lifestyle-related intervention studies aiming to improve the gut microbiota in people with schizophrenia and first-episode psychosis are limited. A 2021 systematic review explored the effect of add-on strategies with known gut-microbial action on total, positive, negative and cognitive symptoms of people with schizophrenia: 21 studies investigated antibiotics, four antimicrobials, and three pre/probiotics [49]. Results were largely negative for these strategies, however there was a paucity of evidence for pre- and probiotic studies. Two recent publications have explored the impacts of fiber and probiotic supplementation on body weight in this population group [50,51]. One publication reported a 12-week intervention supplementing fiber, probiotics, fiber plus probiotics, or placebo found that fiber plus probiotics was superior to all other interventions on reducing weight/BMI and total cholesterol in people with established schizophrenia [50]. The second publication reported on two RCTs in antipsychotic naïve people with first-episode psychosis commencing olanzapine; the first 12-week study found no effect of probiotics on weight gain, whereas the second 12-week study found that probiotics plus fiber resulted in significantly less weight gain compared to the control group [51]. To the authors’ knowledge the current study was the first to explore the impact of lifestyle and life skills intervention on the gut microbiota in people with schizophrenia, suggesting that improvements in α-diversity may be driven by dietary change.

This study has several limitations. First, the results need to be interpreted with caution as for example, the post hoc power analysis for the increase in Shannon diversity was 49% (based on the medium effect size, *d* = 0.6). This was primarily due to the pilot nature of the study which included a small sample size. This also limited the number of analyses explored, i.e., correlation of the 21 differentially abundant taxa with diet quality, and the extent to which we could explore confounding factors appropriate to exploration of the microbiota. Second, recruitment for the patient groups was challenging. Recruitment of people with established schizophrenia receiving clozapine therapy was more successful than people with a first-episode psychosis, however recruitment for both groups was ceased prematurely due to COVID-19 restrictions. The approach to recruiting people with first-episode psychosis and its feasibility requires further exploration. Third, the prospective data lacked a control group reducing the ability to test causality. However, the observed effect will be useful in sample size calculations in future RCTs. Fourth, we were not able to account for some confounding factors such as smoking and the use of additional psychotropic medications. Fifth, this pilot study explored microbial diversity only, future studies should explore functionality [52,53].

This pilot study provides further support for a difference in microbial composition in people with established schizophrenia receiving clozapine therapy and provided preliminary evidence suggesting lifestyle intervention can affect Shannon diversity. Whether this change in Shannon diversity has potential to mitigate the negative side-effects of antipsychotics on cardiometabolic health is yet to be determined. Larger studies using metagenomic sequencing, and controlling for, and understanding the impact of, confounders are needed across relevant population groups: ultra-high risk for psychosis, first-episode psychosis, and established schizophrenia. Well-designed RCTs with sufficient power are needed to determine causality of lifestyle intervention on microbiota changes, and subsequent cardiometabolic outcomes, in this population group.

## Figures and Tables

**Table 1:** Clinical and demographic details of participants.

	First-Episode Psychosis (n = 5)	Established Illness (n = 17)	Total with Psychosis (n = 22)	Matched Controls (n = 22)	Statistical Test	*p*-Value
Female (n, %)	1 (20)	9 (53)	10 (45)	10 (45)	*X*^2^ = 1.69	0.43
Age (mean, SD)	21.8 ± 3.3	44.2 ± 9.7	39.5 ± 14.8	38.7 ± 14.9	*Z* = 118	0.80
Ethnicity (n, %)						
Europid	4 (80)	13 (76)	17 (77)	17 (77)		
South American	0 (0)	0 (0)	0 (0)	1 (5)		
Asian	0 (0)	2 (12)	2 (9)	4 (18)	*X*^2^ = 13.0	0.22
North African/Middle Eastern	0 (0)	1 (6)	1 (5)	0 (0)		
Polynesian	1 (20)	0 (0)	1 (5)	0 (0)		
Other	0 (0)	1 (6)	1 (5)	0 (0)		
Weight (kg) (mean, SD)	60.4 ± 11.1	92.9 ± 21.2	85.5 ± 23.7	81.6 ± 15.3	t (21) = −0.68	0.51
BMI (kg/m^2^) (mean, SD)	20.3 ± 2.8	31.9 ± 6.9	29.2 ± 7.9	27.8 ± 5.0	t (21) = −0.83	0.41
BMI Classification (n, %)						
Underweight	2 (40)	0 (0)	2 (9)	0 (0)		
Normal	3 (60)	2 (12)	5 (23)	9 (41)		
Overweight	0 (0)	5 (29)	5 (23)	5 (23)	*X*^2^ = 6.21	0.29
Obese Class I	0 (0)	4 (23.5)	4 (18)	6 (27)		
Obese Class II	0 (0)	4 (23.5)	4 (18)	2 (9)		
Obese Class III	0 (0)	2 (12)	2 (9)	0 (0)		
Diet Quality	47.0 ± 16.0	51.1 ± 17.3	50.0 ± 16.7	NA	-	-
V02 Submax	47.5 ± 5.0	23.4 ± 6.3	26.6 ± 10.4	NA	-	-
Antipsychotic Medication						
Clozapine (and other antipsychotic)	0 (0)	9 (53)	9 (41)	NA	-	-
Clozapine (only)	0 (0)	8 (47)	8 (36)	NA	-	-
Olanzapine	1 (20)	0 (0)	1 (5)	NA	-	-
Risperidone	1 (20)	0 (0)	1 (5)	NA	-	-
Aripiprazole	3 (60)	0 (0)	3 (14)	NA	-	-
Mood Stabiliser (n, %)	0 (0)	4 (24)	4 (18)	NA	-	-
Antidepressant (n, %)	0 (0)	8 (47)	8 (36)	NA	-	-
Metformin (n, %)	0 (0)	11 (65)	11 (50)	NA	-	-

## Data Availability

Sequencing data have been deposited to the NCBI under BioProject PRJNA896324.

## Supplementary Materials

The following supporting information can be downloaded at: https://www.mdpi.com/article/10.3390/pathogens11111279/s1, Figure S1: Comparison of different alpha diversities using paired samples Wilcoxon test between groups at baseline, Figure S2: Comparison of different alpha diversities using paired samples Wilcoxon test in Established patients with schizophrenia for differences pre- and post-KBIM intervention.
