# Supplementary material for: The Role of the Microbiome in the Metabolic Health of People with Schizophrenia and Related Psychoses: Cross-Sectional and Pre-Post Lifestyle Intervention Analyses
Source: Pathogens. 2022 Nov 1;11(11):1279. doi: 10.3390/pathogens11111279 (PMC9695516; doi:10.3390/pathogens11111279)
Supplement: Supplementary file 1 [file pathogens-11-01279-s001.zip › pathogens-1917038-supplementary.pdf]

Supplementary

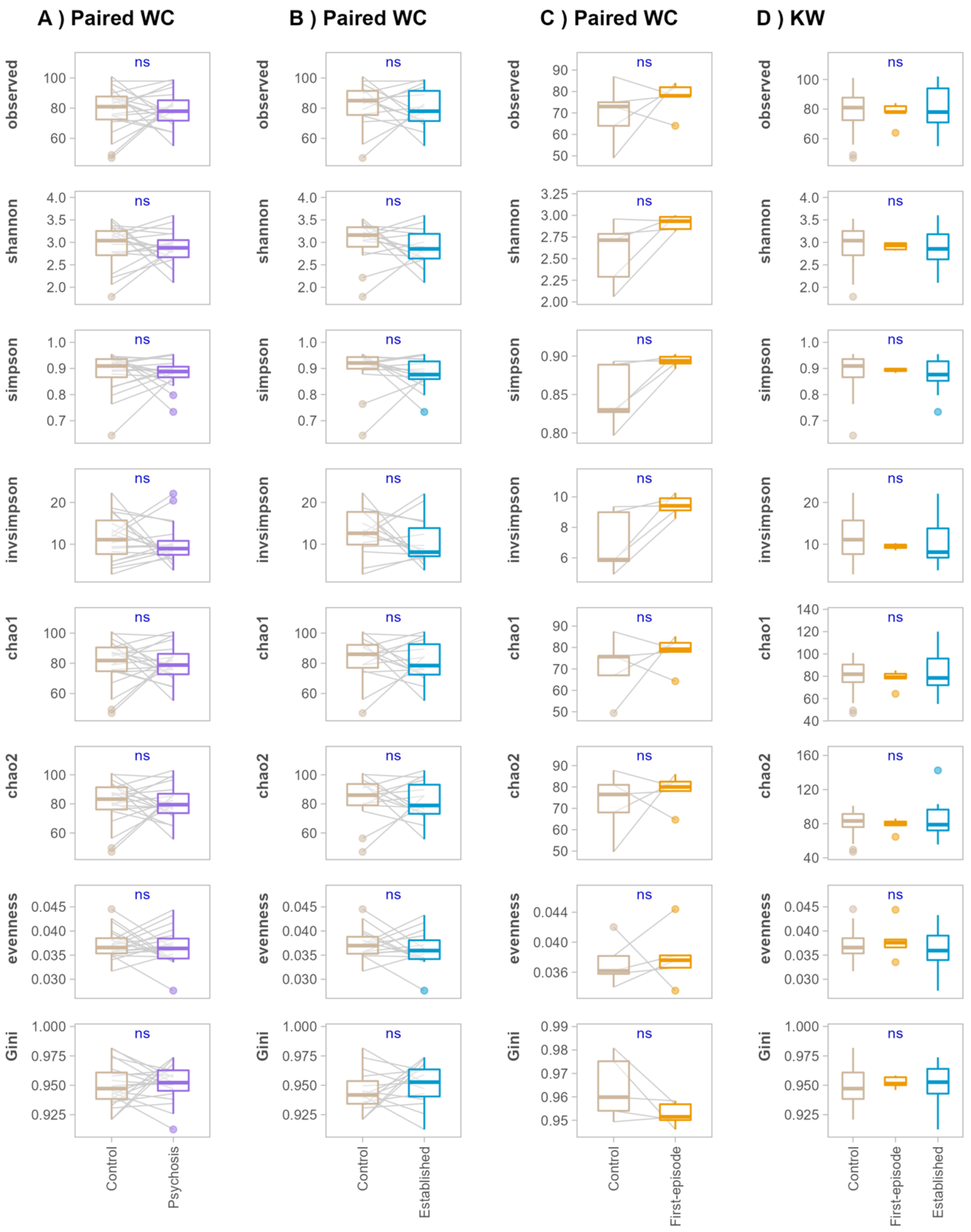

Figure S1. Comparison of different alpha diversities using paired samples Wilcoxon test between [A] Control versus Psychosis that includes both Established and First-onset patients (n=20 of the 22 matched pairs remained after rarefaction normalisation), [B] Control versus Established patients (n=15 of the 17 remained after rarefaction), [C] Control versus First episode patients (n=5 matched pairs) and using Kruskal-Wallis test for testing for any difference across the three groups [D] (unpaired test using all data). No significant results were observed. Abbreviations and symbols - WC: Wilcoxon test; KW: Kruskal-Wallis test; ns: non-significant (p > 0.05).

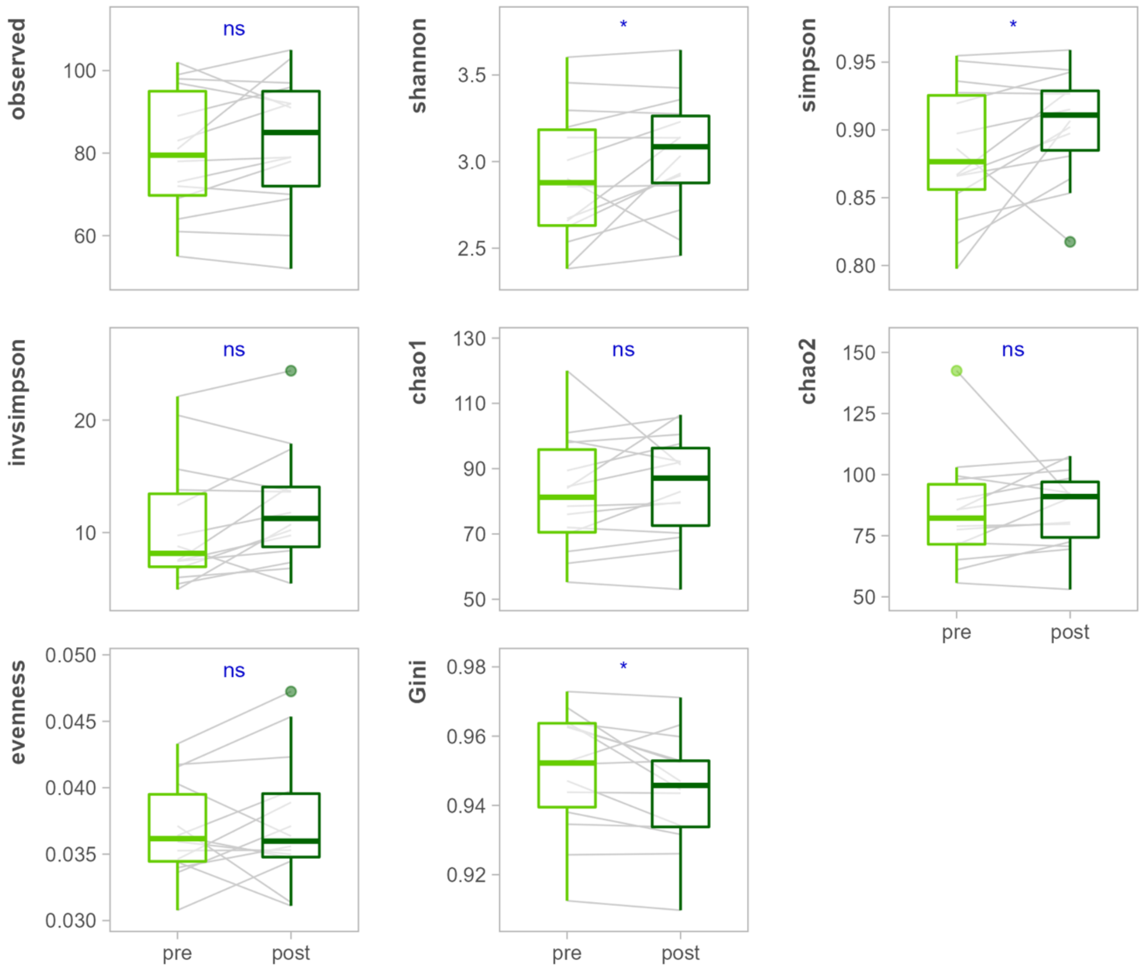

*Figure S2. Comparison of different alpha diversities using paired samples Wilcoxon test in Established patients with schizophrenia for differences pre- and post-KBIM intervention. Of the 17 patients, 14 had samples for both timepoints after rarefaction normalisation. In addition to Shannon diversity showing significant difference, Simpson and Gini were also significant. Abbreviations and symbols - ns: non-significant ($p > 0.05$), * ($p \leq 0.05$).*
